# A method for the identification of guinea pig blood meal in the Chagas disease vector, *Triatoma infestans*

**DOI:** 10.1186/1475-9292-6-1

**Published:** 2007-01-12

**Authors:** Juan Carlos Pizarro, David Lucero, Lori Stevens

**Affiliations:** 1 Department of Biology, University of Vermont, 109 Carrigan Drive, Burlington, VT 04505, USA; 2 Facultad de Bioquímica, Universidad de San Francisco Xavier de Chuquisaca, Sucre, Bolivia

## Abstract

**Background:**

In a SINE-based PCR assay, a primer set specific for guinea pig genome short interspersed elements DNA was used to test the utility of genomic markers for identifying the source of vertebrate blood meals of *Triatoma infestans*.

**Methods:**

The investigation consisted of two assays. In Assay 1, thirty-six insects, collected from the Province of Zudáñez in Chuquisaca, Bolivia were frozen 1–40 hours after feeding, under controlled conditions, on guinea pigs. The species of the vertebrate host was confirmed from dissection of the posterior part of the abdomen of each insect followed by DNA extraction and PCR amplification. Assay 2 investigated whether the technique worked under field conditions. We analyzed the bloodmeal of 34 insects collected from households and peri-domestic structures from communities where wild and captive guinea pigs occur. After collection, the insects were maintained at room temperature for 2 months without feeding and then analyzed.

**Results:**

In Assay 1, each of the 36 insects allowed to feed on guinea pig blood tested positive for guinea pig DNA. The guinea pig DNA was reliably identified in as little as 1 hour and up to 40 hours after feeding. For Assay 2, 8 out of the 34 samples (23%) showed positive results with guinea pig specific primers.

**Conclusion:**

The results in assay 1 demonstrated that DNA from the vertebrate host can be amplified 1–40 hours post feeding from the abdomen of the blood-feeding Chagas disease vector *Triatoma infestans*. The results in assay 2 confirmed that the procedure works on insects collected from households and peri-domestic structures and that the source of a blood meal can be determined at least 2 months post feeding.

## Background

Chagas disease is caused by the flagellate protozoan *Trypanosoma cruzi*, and is transmitted by bloodsucking insects of the subfamily Triatominae (Hemiptera, Reduviidae). At present, estimates indicate that 13 million people are infected with the causative agent, with 3.0–3.3 million symptomatic cases and an annual incidence of 200,000 cases in 15 countries [1]. Post-infection, ten to 20 years of asymptomatic conditions lead to the possibility of cardiac conditions (27%) and probable sudden death, digestive tract problems (6%) and nervous system dysfunction (3%) [2]. There is no vaccine against *T. cruzi*. Treatment with medication is not always successful. Medications work best in the early acute stages, with reduced success up to 10 years post infection, especially in children under 14 years of age [3]. The recommended methods of disease control include screening blood donations, eliminating domestic vector populations by plastering adobe walls and using tile roofs to reduce vector habitat in homes, and spraying infested houses with residual insecticides [4]. These methods, although effective through much of Central and South America, have not been successful in Bolivia where prevalence of human infection and house infestation by the main vector in this area, *Triatoma infestans*, remains high. Interrupting transmission is also complicated because the causative parasite, *T. cruzi*, has multiple mammalian hosts. Although vectors feed on a variety of vertebrate hosts, including mammals and birds, only mammals maintain an infection. Further, persistance of *T. cruzi *among hosts, varies among geographic regions, being influenced by the local domestic vertebrate populations, peri-domestic, as well as possible zoonotic or "wild" vertebrate hosts. In many geographic regions dogs and cats are important host blood sources for domiciliary *T. infestans *[5,6]; however, in the Andean regions of Bolivia and Peru, the domestic guinea pig (*Cavia porcellus*) is also an important reservoir of *T. cruzi *[7,8]. Sylvatic populations of *T. infestans *are only known to occur in the Andean highlands and probably are a major factor in local triatomine population dynamics and reinfestation patterns. Therefore, studies on the ability of vectors to move among habitats and colonize human habitations are important components of disease control because they will help to assess the dynamics governing contacts between humans and vectors [9] and determine the most appropriate strategy for insecticide application. Identification of the source of the blood meal in the digestive tract of the hematophagous triatomines that transmit Chagas disease provides data on host-feeding patterns and preferences in nature. These data provide information about epidemiologically significant disease reservoirs for the most serious human parasitic disease of the Americas in terms of social and economic impact [10].

Historically, blood meal identification of hematophagous insects have been based on antigen-antibody reactions [11,12] searching for antigens present in blood meals using specific antibodies developed by stimulation with the same type of molecule. These methods have proven useful for the identification of many different animal species, but these approaches are labor intensive and the detection limits are restrictive [9].

Polymerase chain reaction (PCR) analysis of species-specific mitochondrial DNA sequences is currently the most commonly used method for species identification. Several mitochondrial DNA genes, such as conserved sequence blocks (CSBs), 16S ribosomal RNA gene and cytochrome *b *(*cytb*) gene, used to study animal evolution [13] can be used to identify bloodmeal sources. The advantage of mitochondrial-based DNA analyses derives from the fact that there are many mitochondria per cell, making it easier to extract sufficient quantities of DNA for reliable PCR amplification [14]. However, species-specific mitochondrial primers are not feasible for the lower levels of taxonomic classification necessary when vectors feed on multiple hosts. Species identification of mitochondrial DNA requires cloning and sequencing multiple clones from each vector to determine the identity of host species fed upon. Recently, the development of PCR assays based on sequences of short interspersed elements (SINEs) has been reported for the detection of species-derived DNA. A series of class-specific (Aves), order-specific (Rodentia), and species-specific (equine, canine, feline, rat, hamster, guinea pig, and rabbit [14], human [15], bovine, porcine, chicken, and ruminant [16]) PCR assays have been designed for the identification of DNA from complex sources.

Repetitive elements can be subdivided into those that are tandemly arrayed or interspersed (for example, mobile elements and processed pseudogenes). Interspersed repetitive elements can be subdivided on the basis of size, with short interspersed elements (SINEs) being repetitive sequences with a length of 70–500 bp that are widespread among eukaryotic genomes [17]. All eukaryotic genomes contain mobile elements, although the proportion and activity of the classes of elements varies widely between genomes. Most mammalian SINEs have appeared within the past 65 million years and are thought to have been spread throughout each genome via an RNA-mediated duplication process termed retroposition [18]. Their retrotransposition depends on reverse transcriptase and endonuclease activities encoded by partner LINEs (long interspersed elements). More than 30 families of SINEs have been characterized in mammalian genomes, comprising 4600 extant species. Every mammalian order has a significant number (> 100,000) of characteristic mobile elements providing specific tags that can be used in a PCR to amplify specific sequences from mixed DNA sources [14]. The guinea pig ID3-SINE/ID (AF312680, [14]) used in this assay has an estimated 200–3000 copies in the guinea pig genome [19]. Species specific primers have been developed and used for identification of biomaterials from complex sources, thus we tested its feasibility for the abdominal content of triatomines.

Reliable and efficient determination of feeding behaviour patterns of Chagas' disease vectors is important for disease epidemiology assessment. Using species-specific primers in the PCR has the potential to decipher each component of a multi-species DNA mixture. Because understanding vector movement and feeding preferences are important to the success of vector spraying programs we experimentally tested the ability of PCR to detect vertebrate blood in the abdomen of *T. infestans *from 1–40 hours after feeding (assay 1) as well in triatomines collect from communities in the Department of Chuquisaca, Bolivia where wild and domestic guinea pigs occur (assay 2).

## Results

**Figure 1:**
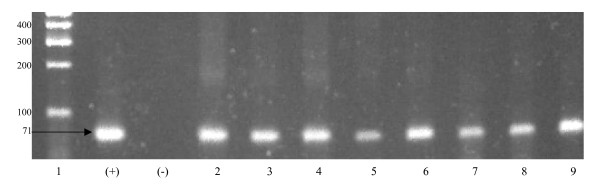
Ethidium bromide stained agarose gel of PCR amplified SINE guinea pig DNA. Template DNAs for each lane were: 1 100 bp size standard, (+) guinea pig positive control, (-) PCR negative control (no template), 2 – 9 1, 3, 9, 15, 18, 24, 30 and 40 hours after feeding. Value on the left is in basepairs (bp).

The results of assay 1 verified that the primers successfully amplified DNA extracted from the abdomen of one adult and two nymphs of *T. infestans *sampled 1, 3, 5, 9, 11,13, 15, 18, 24, 30, 35, 40 hours after feeding. No amplification was observed in the negative controls. The fragment size of the experimental and positive control reactions were as expected for guinea pig (Figure 1). Comparison of the sequence of the PCR products to those in GenBank confirmed that the amplified product was guinea pig DNA. The results of the assay indicate that at most 1 hour is needed for host DNA to reach the abdomen where it was still present after 40 hours.

The results of assay 2 revealed guinea pig DNA in 23% (8 out of the 34 bugs) of the vectors collected from domestic and peri-domestic structures. There was no difference in the likelihood of amplification for insects from different habitats or life stages. These DNA extractions have subsequently been used for insect microsatellite analysis (unpublished data), indicating that the negative samples were not simply results of PCR inhibition. In addition, some of the negative samples were spiked with 0.3 ng guinea pig DNA and subsequent PCR with guinea pig specific primers showed amplification in all cases.

## Discussion

The sensitive and reliable identification of blood from complex sources has been an important objective in studies involving the ecology of infectious disease. With respect to *T. infestans*, previous studies of species identification used anti-sera specific reactions whose specificity is limited to genus [5,20]. There are several advantages to PCR over serologic methods. First, PCR is very sensitive requiring only a small amount of biological material that need not be fresh or kept frozen. In addition, when amplicons are relatively small, even highly degraded DNA templates may be amplified [14]. When primers are directed to multi-copy nuclear DNA, or DNA from organelles such as mitochondria that are abundant in many tissue types, such assays can be highly sensitive. Another advantage is that PCR based methods can use simple agarose gel analysis. We developed a PCR based assay based on a nuclear DNA sequence with high copy number. Reagents are commercially available, the assay has relatively low cost for analyses on a large-scale and minimal equipment requirements, so that laboratories with modest resources are able to perform these assays.

In this study we evaluated a species-specific (guinea pig) PCR assay for the identification of DNA extracted from the blood meal of the heamatophagous Chagas' disease vector *T. infestans *based upon intra-SINE PCR amplification of genome-specific interspersed repetitive elements. For assay 1, 34 second, third, forth and fifth instar nymphs and two adult males collected from a goat corral in Jackota were analyzed; those for assay 2 were 31 insects from domestic and peri-domestic habitats in Zurima and three specimens from the same goat corral in Jackota examined in assay 1. It is likely that most insects in assay 1 had previously fed on animals other than guinea pig, thus, the DNA extraction samples obtained after feeding with guinea pigs in addition to guinea pig and *T. infestans *DNA, may have had additional vertebrate DNA as well as DNA of the parasite, *T. cruzi*. Assay specificity was demonstrated by amplifying guinea pig DNA from this mixed-species template followed by confirmation through DNA sequencing.

Because we were able to detect a specific blood source from a mixture of DNA, the technique should be capable of classifying mixed blood meals using a multiplex protocol with different species-specific primers. A study using a heteroduplex assay for identifying bloodmeals in the Chagas disease vector *Triatoma longipennis *compared cytochrome *b *DNA conformations with patterns from control standards [9]. The authors stated that mixed blood meals were likely in at least 6% of the insects they examined; however, the heteroduplex assay is unable to make identifications in such cases. Species-specific PCR primers have the advantage that they can be used to specifically amplify a particular DNA of a natural mixed blood meal. Having reliable and inexpensive primers already developed for birds and mammals that constitute the main source of food for the vectors of Chagas' disease, it should be possible to conduct a complete assessment of the feeding profile of triatomines. In practice, the ability to detect and characterize a mixed blood meal will depend in the relative concentration of the DNA from each host present in the blood meal, as well as "stochastic processes, such as PCR selection and PCR drift, which can affect the relative efficiency of the PCR amplification of two or more targets in a mixed template" [11].

The amplification of the genome-specific SINE-guinea pig assay is based on a marker with only 71 base pairs enhancing the ability to identify DNA from highly degraded samples. This is an advantage over other techniques, especially for old samples or samples from dead insects. Previous studies using PCR to detect *T. cruzi *in Triatomine vectors found that the stomach was more often negative whereas intestine and rectum samples showed much less inhibition to PCR, reflecting either differences in the distribution of *T. cruzi *or PCR inhibition [21]. The method presented here, of extracting samples from the rectal and posterior part of the abdomen using the DNeasy kit (Qiagen, Valencia CA) did not display PCR. inhibition problems. We demonstrated that the samples scored as negative for guinea pig blood in assay 2 could amplify DNA by "spiking" and subsequently amplifying guinea pig DNA; this procedure amplified DNA in all samples tested.

In this study, the guinea pig specific assay consistently amplified the target DNA from nymph and adult *T. infestans *from one to 40 hours after feeding. Because Chagas disease vectors are long-lived, they are likely to feed on several hosts. Detection of feeding over weeks or months can provide important information on feeding preferences and host movement. Our results from assay 2 demonstrate that DNA remains in the digestive system of insects for at least two months. Heteroduplex analysis in *T. longipennis *found DNA traces of blood meals in vectors up to 30 days post feeding [9]. Assay 2 extends the upper limit of detecting blood meals to two months. Although a positive result indicates past feeding on a guinea pig, for a negative result further study is needed to determine whether guinea pig DNA is absent or is due to the loss of signal over time and the rate at which, a blood meal is eliminated or degraded to the point it can no longer be detected.

Assays 1 and 2 included blood meals from domestic guinea pigs from 2 different regions and different developmental stages in the Chuquisaca area. In all cases 2% agarose gel electrophoresis with ethidium bromide staining did not detect differences in the sizes of the DNA fragment of all positive samples. Genbank DNA sequences from both published and unpublished studies and sequences from our PCR products were identical and confirmed the identity of our PCR product as *C. porcellus*. Domestic guinea pigs, a common regional food, are maintained in houses. Wild guinea pigs are native to this area and often burrow in rock piles within goat corrals. We were able to detect guinea pig DNA in insects collected both inside and outside of houses. Because wild and domestic guinea pigs are the same species, with identical sequences in the region of DNA examined, we were not able to differentiate whether there is active exchange of triatomines between these two habitats or they are feeding on their own habitat hosts.

The potential of DNA-based molecular markers to detect the source of arthropod blood meals has recently been recognized [22]. These techniques are likely to contribute major breakthroughs in our study of the transmission dynamics of vector borne diseases. The development of species specific markers that can detect feeding at a minimum of two months post feeding presents a valuable tool for tackling Chagas disease, a socially and economically devastating infectious disease in South America [10].

## Conclusion

In this study we have evaluated a species-specific (guinea pig) PCR-based assay to identify the souce of the vertebrate blood in abdomen of the heamatophagous Chagas disease vector *Triatoma infestans*.

We have demonstrated that DNA from the vertebrate host can be amplified 1–40 hours post feeding from the abdomen of triatomines. This study also confirmed that the procedure works on insects collected from households and peri-domestic structures and that the source of a blood meal can be determined at least 2 months post feeding.

This assay possesses the unique ability to analyze components of a multispecies DNA sample. It provides a simple, reliable DNA-based test for the detection of guinea pig blood present in feces and the rectum content of the reduviid vector of Chagas disease.

## Methods

### Sample collection

The study consisted of two parts. Assay 1 involved a controlled laboratory experiment to assess the utility of the PCR to identify guinea pig blood up to 40 hours after insect engorgement. Thirty-four second, third, forth and fifth stage nymphs and two adult males were collected from a goat corral in Zudañez, Department of Chuquisaca, Bolivia. They were maintained, unfed, in plastic containers with folded filter paper at ambient temperature (~17°C).

After three months the insects were allowed to feed on guinea pig blood by placing five insects in each of seven cages containing a guinea pig. Guinea pigs (Class: Mammalia ; Order: Rodentia; Family: Caviidae; Genus and species: *Cavia porcellus*) weighed approximately 400 grams. Cages were covered with a dark cloth and maintained in quiet surroundings while the experiment was conducted. After one hour, all insects were recaptured alive. Three insects were killed at this time, and the remaining were held in groups of three per plastic container until the designated sampling time (1, 3, 5, 9, 11,13, 15, 18, 24, 30, 35, 40 hours after feeding) at which time insects were killed by placing in 96% ethanol. Each group of three insects consisted of 2–3 different nymphal stages or two nymphs and one adult. Insects were sent to Burlington, Vermont, USA via express courier where they were stored at -20°C until analysis. This protocol was approved by the Universidad de San Francisco Xavier de Chuquisaca Animal Research Committee and follows internationally recognized guidelines.

For assay 2 we examined 20 third, fourth and fifth stage nymphs and 14 adult *T. infestans *collected in 2004 from domestic and peri-domestic structures in Zurima and a corral in the community of Jackota, Department of Chuquisaca, Bolivia. Insects were maintained as described above for two months then killed in 96% ethanol and sent to Burlington, Vermont, USA were they were stored at -20°C until analysis.

### DNA extraction

A new razor blade was used to collect 25 mg of tissue from the posterior part of each insect's abdomen. The cut was made as close to the posterior as possible to avoid the stomach. Subsequent DNA extraction used the DNeasy kit (Qiagen, Valencia CA) following the protocol for animal tissues with 24 hour lysis. DNA concentration was measured using a Nanodrop 1000 spectrophotometer (Nanodrop, Bethesda, MD).

### PCR amplification

The PCR amplification produced a 71 bp fragment using primers specific to guinea pig multi-copy nuclear DNA short interspersed elements (SINEs): 5'-GGG ATT TAG CTC AGT GGC ATA AG-3', 5'-ATT GGT ACC GGG GAT TGA ACT-3', (Repeat element ID3 SINE/ID; Accession no. AF312680 [14]).

Reactions contained 10 μM of each primer, one Ready-To-Go PCR bead (Amersham Bioscience, Piscataway, NJ) and molecular biology grade water to 25 μl. We added 50–100 ng DNA to each reaction, however because DNA extracted from vectors usually contained at least two types of DNA (e.g., insect + guinea pig) we could not quantify the amount of guinea pig DNA in each reaction. Each sample was subjected to an initial denaturation of 1 min. at 95°C followed by 30 cycles of denaturation at 95°C for 30 s, 60°C to anneal for 30 s and 30 s of extension at 72°C. Amplicons were chromatographed on a 2 % agarose gel with a 100 bp size standard, stained with ethidium bromide and visualized using UV fluorescence. Positive controls using DNA extracted from the liver of laboratory guinea pigs confirmed the ability of primers to amplify target DNA, negative controls had no DNA.

## Competing interests

The author(s) declare that they have no competing interests.

## Authors' contributions

JCP: participated in the conception and design of the study, carried out the sample collection, DNA extraction PCR analysis and drafted the manuscript. LS: participated in the conception and design, helped optimize the extraction and PCR analysis and helped to draft the manuscript. DL: carried out the PCR-based detection assay. All authors read and approved the final manuscript.
